# Active Mobility and Environment: A Pilot Qualitative Study for the Design of a New Questionnaire

**DOI:** 10.1371/journal.pone.0168986

**Published:** 2017-01-04

**Authors:** Franck Hess, Paul Salze, Christiane Weber, Thierry Feuillet, Hélène Charreire, Mehdi Menai, Camille Perchoux, Julie-Anne Nazare, Chantal Simon, Jean-Michel Oppert, Christophe Enaux

**Affiliations:** 1 Laboratoire Image, Ville, Environnement, Department of Geography, Strasbourg University, Strasbourg, France; 2 Laboratoire Image, Ville, Environnement, CNRS, Strasbourg, France; 3 Paris 13 University, Sorbonne Paris Cité—Equipe de Recherche en Epidémiologie Nutritionnelle, U1153 Inserm, Inra, Cnam, Centre de Recherche en Epidémiologie et Biostatistiques; CRNH IdF, Bobigny, France; 4 Lab-Urba, Urbanism Institute of Paris, Department of Geography, Paris-Est Créteil University, Paris, France; 5 CARMEN, INSERM U1060/Lyon 1University/INRA U1235, Lyon, France; 6 Department of Nutrition, Institute of Cardiometabolism and Nutrition (ICAN), Pitié-Salpêtrière Hospital (AP-HP), Pierre et Marie Curie University—Paris 6, Paris, France; Peking University, CHINA

## Abstract

It is generally accepted that active mobility, mainly walking and cycling, contributes to people’s physical and mental health. One of the current challenges is to improve our understanding of this type of behaviour. This study aims to identify factors from the daily-life environment that may be related to active mobility behaviours, in order to design a new questionnaire for a quantitative study of a large adult population. The new questionnaire obtained through this pilot study combines information from interviews with existing questionnaires materials in order to introduce new factors while retaining the factors already assessed. This approach comprises three stages. The first was a content analysis (Reinert method) of interviews with a sample of participants about daily living activities as well as mobility. This stage led to a typology of factors suggested by interviews. The second was a scoping review of the literature in order to identify the active mobility questionnaires currently used in international literature. The last stage was a cross-tabulation of the factors resulting from the written interviews and the questionnaires. A table of the inter-relationships between the interview-based typology and the questionnaires shows discrepancies between factors considered by the existing questionnaires, and factors coming from individual interviews. Independent factors which were ignored in or absent from the questionnaires are the housing situation within the urban structure, overall consideration of the activity space beyond the limits of the residential neighbourhood, the perception of all the transportation modes, and the time scheduling impacting the modes actually used. Our new questionnaire integrates both the usual factors and the new factors that may be related to active mobility behaviours.

## Background

It is currently admitted that active mobility, mainly walking and cycling, substantially contributes to the population’s physical and mental health [1,2,3]. According to socio-ecological models of health-related behaviour, both social and physical environmental characteristics influence active mobility in combination with individual-level factors [4,5].

Several tools are used to assess environmental factors that influence active mobility at the population level. Geographical information systems (GIS), which have recently been introduced into health research, coupled with population censuses, allow us to describe these environments in a normative way within the limits of objective, administrative divisions [6,7]. Questionnaires remain an instrument of choice in large population studies to assess residential or working environments. But social and spatial dynamics at the population level imply that the role of behaviour-related factors associated with behaviours such as active mobility is undergoing changes. For example, today’s daily activity places go well beyond the neighborhood of residence thereby reducing its importance in the understanding of travel behaviours. It is therefore necessary to regularly re-assess tools and/or design new instruments.

Environmental factors potentially associated with active mobility can be identified through various sources such as existing literature or expert statements, or through questions asked to residents themselves. Previous research has identified a number of environmental factors consistently associated with active mobility. The literature (theoretical, quantitative and qualitative) resulting from this research identifies the following main elements: built or physical environment factors including land use, accessibility to shops and green space, transportation systems and networks, architecture, sidewalks, traffic, street lightning, and crime rate [8,9]. Social environment factors include income, education and social characteristics [10,11]. This literature is often used along with experts’ contributions [12,13], or more pragmatically, with existing questionnaires or portions of questionnaires [14]. The third resource is based on the results of focus groups or individual interviews to identify environmental factors thought to be important [15].

Combining these three sources of information is important to design new questionnaires focused on the environmental influences on active mobility. On the one hand, data obtained through existing literature or experts’ advice depends on our current knowledge. On the other, individuals’ opinions collected at focus groups or individual interviews allow us: i) to identify new factors previously unaccounted for to understand active lifestyles, ii) to directly assess the evolution of factors involved in active lifestyles.

The aim of the present work was to design a questionnaire to enrich active mobility research with new potentially influential factors, which could be used through self-report in large-scale population studies in France. We assumed that direct recourse to individuals can identify new potentially important factors, and thus fill potential gaps in this research field. First, we describe our methodological approach and the factors resulting from a content analysis of interviews with a sample of French individuals dealing with daily living activities. Here, activities are defined as what individuals do at home and away from home. This broad definition of activities fits with the theoretical activity-based approach which states that the movements of an individual, in particular the use of transportation modes, depends on a complex interplay of decisions involving all of his daily activities [16,17]. In order to draw a list of factors likely to influence active mobility, we took into account all the factors presented by interviewees as enhancing or restricting daily life activities. Secondly, we compare the list of factors resulting from the analysis of the interviews with data from existing questionnaires identified through a scoping review of the literature on active mobility. As we wanted to identify the gaps in existing questionnaires without missing any of the factors arising from the interviews, our review targeted more identifying these gaps than assessing their quality [18].

## Design and Methods

**Fig 1:**
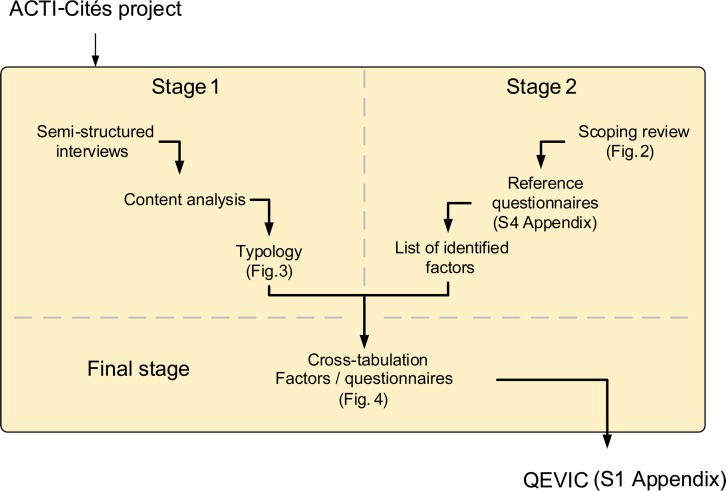
Methodological approach.

Our methodological approach, summarized in Fig 1, was divided into 3 stages. Stages 1 and 2 aimed at identifying factors in the speech of individuals and in existing literature respectively. In the final stage, we compared the factors of the first two stages. The 3 stages led to the design of the QEVIC (Questionnaire on daily-life environment) shown in S1 Appendix as a supplement to this article.

### Stage 1

#### Semi-structured interviews

Semi-structured interviews were conducted in French with 9 adults (age > 18 years) living in the metropolitan area of Strasbourg (France). We selected subjects with differing profiles in terms of sociodemographic, socioeconomic and residential origin. The interviewees were 4 women and 5 men, aged 39.2 ± 13.8 years (mean±SD), differing in household types (3 single people, 3 couples without children and 3 couples with children), in socioeconomic categories (3 blue-collar workers, 3 white-collar workers and 3 executives) and types of residential neighbourhoods (3 in the city centre: centre or around the centre, 3 in the suburbs: outlying or inner suburbs, 3 around the urban area: village or small urban centre). This series was a convenience sample recruited among acquaintances of other members of the Faculty of Geography, University of Strasbourg. The interviewer did not know the interviewees. Contact was made by telephone. The face-to-face interviews took place at locations that were chosen by the interviewees (home, workplace or laboratory) and no other person was present during the interview. The interview guide is shown in S2 Appendix.

The interview protocol focused on daily activities (including travel) performed during a usual week by discussing their type, the reasons and restrictions to these activities, as well as their geographical and social context and timeframe, in order to assess all the factors potentially related to active mobility. It was decided not to refer to active lifestyle and active travel as ‘the norm’ during the interviews, in order to minimize possible social desirability bias [19]. To limit recall errors, the interview protocol defined an order to discuss activities: at home, in the neighbourhood, beyond the neighbourhood (city, region, etc.). These interviews terminated when the speech of the subjects became redundant. The interviews were recorded (29.3 ± 6.9 min) with the consent of all the subjects, transcribed verbatim and combined to form a single corpus in French, for later content analysis.

This research is in line with the general ethical protocol for scientific research of the Strasbourg University, and was approved by the Ethics Committee of Faculties of Medicine, Dentistry, Pharmacy, of Nurses Schools, of Physiotherapy, of Maieutic and Hospitals of Strasbourg, France. In accordance with the study protocol, the records and the corpus were erased after analysis to respect the respondents’ privacy.

#### Analysis of interviews

We used Reinert’s method for text analysis [20,21]. It consists in a hierarchical descending classification of text segments of the corpus, *i*.*e*. on complete sentences or parts of sentences separated by punctuation with a maximum length of 40 words. This involves classifying text segments (n = 1,017) according to lemmatized active forms in the whole of the corpus. Lemmatization means reducing all variants of the same word to a single root: for example, a verb is used in its infinitive form. A special case of lemmatization concerns proper nouns related to places, such as the names of parks. In this case, they are replaced by a generic word like “park”. The active forms consist in important words (*i*.*e*., those which carry a strong meaning) which are nouns, verbs, adjectives and adverbs. Words which are used to construct sentences, such as prepositions, pronouns, conjunctions and auxiliaries, were ignored. They were assimilated into descriptive forms, although the final interpretation could be based on these forms to qualify text segments groupings in the corpus.

The analytical procedure of Reinert’s method includes three successive steps. The first is a Factorial Correspondence Analysis [22] based on the profile of text segments according to the presence or absence of active forms: the first factorial axis gives the main direction to distinguish sentences with a positive correlation (group 1) or negative correlation (group 2). The second step is an optimization of these two groups by successive permutation of the sentences they contain in order to maximize the second-order moment of the partition (*i*.*e*., intra-class variance minimization). The third step consists in eliminating from each group the most characteristic active forms of the other group (within the meaning of chi^2^). These three steps are repeated starting from the group containing the largest number of sentences, thus leading to the development of a hierarchical tree. The analysis of our corpus was carried out using Iramuteq v0.7, an open-source text mining freeware developed at the University of Toulouse, France (http://www.iramuteq.org). Analyses were performed on the original corpus in French. The results were translated into English afterwards.

#### Identification of factors resulting from the interviews

The hierarchical classification obtained by Reinert’s method eventually leads to increasingly homogeneous classes as to the active forms which they are associated with. Each class is characterized by the percentage of the active form, by the chi^2^ of the active form class-membership, and by its statistical significance (p<1%). The interpretation of the classes is based on these criteria. For example, the most significant active forms of a class may concern words relating to sport activities, words reflecting the sport activity performed by the parents, and words related to the importance of current social relationships during these activities. It would mean, and this can be verified easily by consulting the text segments of the class, that sport activities are strongly connected to a parental model predisposition for sport, but also to the nature of social relationships forged in pairs, this being a motivation to engage in the practice of the sport.

The choice of the number of typology classes depends on the study goal. Here, we were concerned with creating a questionnaire based on environmental factors influencing active mobility. The selected criterion is that, by the active forms which are significantly associated with it, each class should imply a specific aspect: for example, the street layout or the ambience of a neighbourhood. Specifically, typologies based on a precise division (*i*.*e*., a large number of classes) were explored in order to favour a single factor for each class.

### Stage 2

#### Searching for existing environmental questionnaires to study active mobility

The list of environmental questionnaires on active mobility was drawn from a scoping review of the literature. We used search terms related to the environment (“physical environment”, “built environment”, or “social environment”), and to active mobility (“active mobility”, “active transportation”, “walking”, “cycling” or “bicycling”). The full list of keywords and the full requests for each database are shown in S3 Appendix. Web databases searched were PubMed, Web of Science and Transport Research International Documentation (TRID). The search of English-language references was limited to European studies of human mobility published between 01/01/2009 and 31/05/2015.

**Fig 2:**
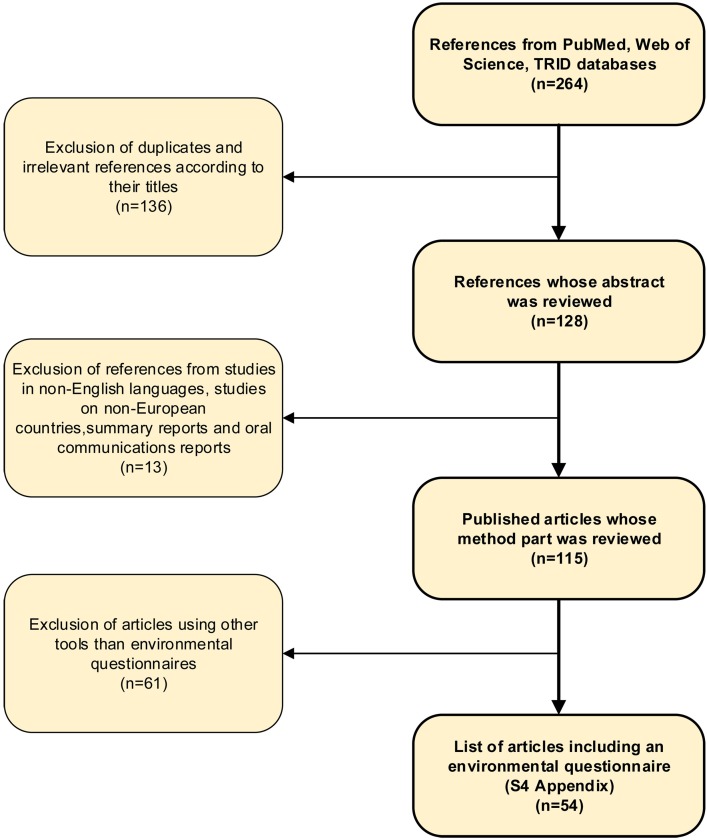
Scoping review’s flowchart.

Fig 2 shows the flowchart for the review of questionnaires on active mobility. Among 264 references from a search on PubMed, Web of Science and TRID, a first selection was made to exclude duplicates and references whose title indicated that they were irrelevant to our query. The remaining references (n = 128) were analysed in order to exclude languages other than English, works relating to non-European countries, reports, and oral communications. The methodological parts of the remaining articles (n = 115) were analysed so that finally, only those based, or partly based, on an environmental questionnaire were selected. Questionnaires intended for specific populations (children, children and active modes of transportation for commuting to school; elderly people) were retained.

S4 Appendix shows the selected articles (n = 54) organized in a table according to the questionnaires they rely on. References under the questionnaires names link to the references used, either to the questionnaires themselves (if available), or to articles describing their content. Among the 27 questionnaires selected, 7 are identified by their names. When several versions of the same questionnaire were available, we analysed the extended version. Twenty questionnaires were not explicitly named and were referred to as “No Name 1 to 20”.

#### Data extracted from environmental questionnaires

Each questionnaire in S4 Appendix was analysed to determine the factors related to the various items it contains. Each item of the questionnaire was considered separately, to be associated with a factor that could potentially affect active mobility. For example, several questionnaires included items relating to shops and services in the residential neighbourhood. Individuals were asked about these shops and services, the time they need to go there on foot, and how often they visit them. Thus, we considered that these items referred to three different factors: namely, the presence of shops and services, the time needed to reach them, and places visited.

### Final stage

The final stage was to combine factors identified by the analysis of the interviews (as reference) with factors identified by the analysis of the questionnaires through cross-tabulation. When a factor in a questionnaire corresponded to a factor resulting from the typology, it was marked with a greyed box in the cross-tabulation. When a factor in a questionnaire did not appear in the typology, an additional column was added in the cross-tabulation and ticked for the questionnaire. In the figure resulting from this cross-tabulation analysis, rows correspond to the questionnaires identified (n = 27) and columns include all typology factors as well as the factors from the questionnaires missing in the typology (n = 34).

## Results

The main results concern the typology (stage 1), and the cross-tabulation (final stage).

### Stage 1: Typology

**Fig 3:**
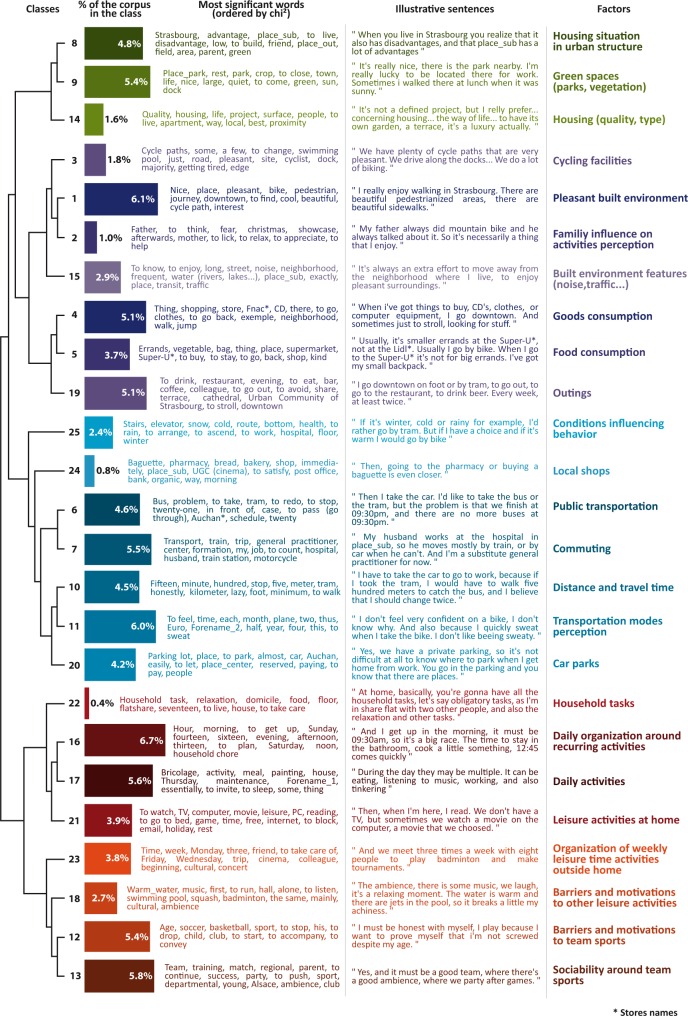
Typology from interviews. Each line depicts a factor from the interviews analysis. The hierarchical tree represents the proximity of these factors.

The final typology obtained by analysis of the interviews is shown in Fig 3. It comprises 25 classes including from 0.4% to 6.7% of all the text segments of the corpus (n = 1017). These 25 classes refer to 25 factors potentially relevant for daily activities practice. These 25 factors are structured in the hierarchical tree as two broad themes. The first (green, purple and blue colours) is the activity space, *i*.*e*., all the places visited in everyday life to carry out various activities and the routes used to reach these places [23]. The second theme (orange and red colours) is the activity timeline which reflects the way in which activities are organized within the constraints of available time [24].

#### Activity space factors

The activity space includes 17 factors. The first three are the housing situation (class 8), green spaces (class 9), and the quality/type of residence (class 14) which put housing in its context (neighbourhood and urban structure). To carry out activities of consuming goods and food as well as going out (classes 4,5 and 19), the key factors are the characteristics of the built environment related to cycle paths (class 3) and the aesthetics of activity places and spaces adjacent to routes travelled on foot or by bicycle (class 1), as well as the advantages (access to water, proximity, etc.) and disadvantages (traffic, noise, distance, etc.) of activity environments (class 15). The social environment also occurs in this theme with family influence on the perception of activities (e.g. cycling) (class 2).

The transportation system and trip appear particularly strongly connected with stores and local services (class 24), and commuting between work and home (class 7). The use of any particular transportation mode depends on various conditions (weather, health, etc.) which affect that mode (class 25). Travel is also mentioned in relation to the distance between activity places, and the time taken by each transportation mode to reach them (class 10). In addition, the perception of transportation modes (class 11) and parking facilities (class 20) both appear to influence transportation mode choice. As far public transport (class 6), schedules are mentioned as inadequately addressing time constraints.

#### Temporal organization of activities

The topic of the activity timeline can be divided into two parts. The first concerns mainly the time of the day (class 16) and involves repetitive activities such as housework (class 22), but also all other daily activities such as meals and sleep (class 17), or leisure activities carried out at home (class 21). The second concerns specific leisure activities during the week carried out away from home, and in particular, sports. There appears to be an organizational factor (class 23), but also constraints and motivations which are different for team sports (class 12) and other leisure activities (class 18). The practice of team sports appears to depend on activity-oriented social interactions (class 13).

### Final-stage: Cross-tabulation

**Fig 4:**
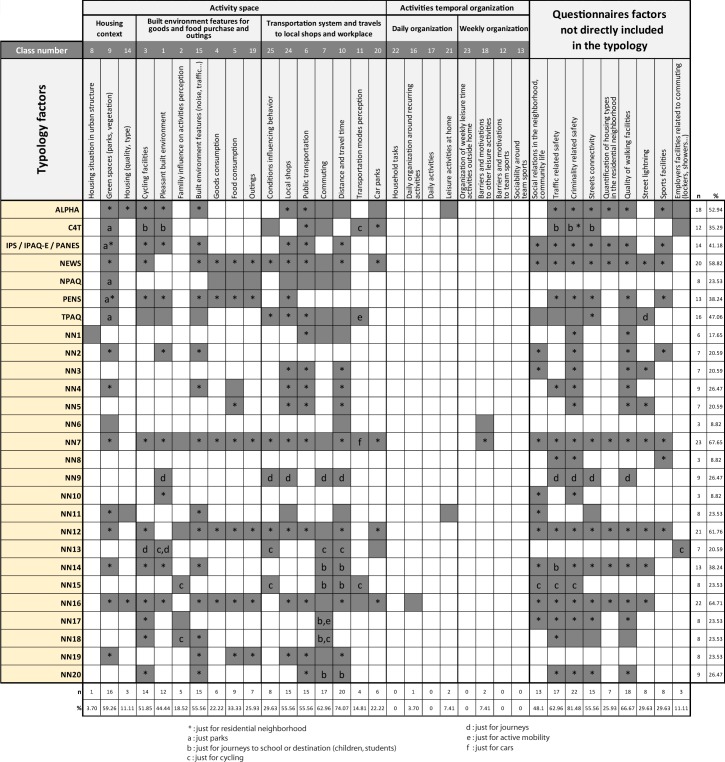
Cross-tabulation of factors and currently used questionnaires.

The cross-tabulation of inter-relationships between the typology and the questionnaires (Fig 4) shows a discrepancy between those factors resulting from the text analysis, and those obtained from the questionnaires. No questionnaire covers all typology factors, and conversely, typology does not explicitly mention some factors, such as social networks and community life, traffic-related safety, crime, street connectivity, the numbers of different types of buildings in residential neighbourhoods, the quality of sidewalks, sports facilities and equipment provided by employers (changing rooms, showers, etc.), as an entirely separate class. The questionnaires which integrate a majority of factors are those that integrate standard questionnaires (*e*.*g*. NEWS or ALPHA) and additional questions that take account of additional factors, such as NN7, NN16 and NN12. The latter include 67.65%, 64.71% and 61.76% of the factors identified in the literature and the interviews respectively. NEWS and ALPHA include 58.82% and 52.94% of these factors respectively.

#### Activity space

Activity space factors the least frequently taken into account by the questionnaires are of various natures. Dealing explicitly with the built environment, the housing situation within the urban structure (centre, suburbs and outskirts) often implies a specific land use in the residential neighbourhood (functions and diversity), as well as particular accessibility to urban amenities and various transportation systems. Only one questionnaire (NN1) takes account of this. In the text analysis, housing quality refers especially to the housing surface and outdoor areas (garden and terrace) in the context of a single house purchase project. The urban structure makes only possible the completion of such a project in certain outlying areas. The location selection for the new house could thus be detrimental to active mobility. The social environment, in particular the family influence, is still not taken into account extensively by the questionnaires, except for studies on children.

Transportation modes allow the use of the urban environment (built and social) which reflects the activity space of individuals. Nevertheless, it appears that the way in which such modes are perceived is considered in only 5 questionnaires, and without a comprehensive overview of transportation systems covering all modes, networks and parking. This lack of a comprehensive view in the questionnaires is particularly obvious from the viewpoint of the conception of the activity space. Such a conception prioritizes many environmental characteristics of the residential area (see * in Fig 4), often at the expense of travels and characteristics of destinations outside that area, whatever the reason for traveling to them.

#### Timeline

Factors related to the organization of available time identified from the text analysis just appear a few times in the questionnaires. ALPHA and NN11 take account of leisure activities at home, whereas NN6 and NN7 take account of other leisure activities constraints and motivations. However, the use of transportation modes, particularly active modes, is related to doing or not doing an activity, and to the management of those activities (time, place, duration, with whom, their position in the timetable), whether they are done sporadically or routinely [25]. The management of the activity timeline does not directly depend on environmental factors (built and social), but rather on personal factors which are potentially important for understanding the use of transportation modes.

#### Additional factors

Several factors do not appear directly as a specific typological class, although they are generally present in the text corpus and correspond to specific questions in some questionnaires. The factors related to road traffic (17 questionnaires), street connectivity (15 questionnaires), and sports facilities in the neighbourhood (8 questionnaires) are mentioned in the text corpus (see Fig 3 “most significant words”), but their frequency is too low to constitute an entirely separate class. Other factors are absent from the text corpus, such as the proportion of different types of homes in the neighbourhood (7 questionnaires), sidewalks (18 questionnaires), street lightning (8 questionnaires), specific equipment (changing rooms, showers, etc.) provided by the employer at the workplace (3 questionnaires), social relationships in the residential neighbourhood and community life (13 questionnaires) and crime in the neighbourhood (22 questionnaires).

## Discussion

### Main results of the study

This study explores the joint use of interview and scoping review approach to identify potential factors related to active mobility. We will discuss both the thematic and methodological aspects of our research.

From the thematic point of view, the typology from the interviews clearly shows that a large number of factors are potentially important to increase our understanding of active mobility in adults. If factors relating to environment are integrated into the available questionnaires, it appears that the built environment is taken into account in a restrictive way i) by concentrating on the residential area, and ii) by the absence of a factor reflecting the situation of the place of residence within the urban structure. Therefore, the whole activity space [23, 26, 27] should be considered from the viewpoint of the environment (built and social), especially since active travel may take place around activity sites previously reached by some type of motorized transportation mode [28]. For this reason, it is essential to take account of factors allowing us to define all transportation systems (cars, public transport, bicycles and walking), not only those associated with active mobility. This definition should take account of how all transportation modes are perceived, since this is an important factor of modal behaviour in transportation research [29].

An interesting finding in the present study is that the way activities are carried out seems strongly related to individuals’ time organization. This relationship is particularly strong for travel, as interviewees associate mode choice to their time constraints. These personal factors of time organization and perception of transportation modes have so far been ignored in existing questionnaires. Although these personal factors are distant from the themes usually included in environmental questionnaires, our finding suggests the need for the combined use of environmental questionnaires and questionnaires dealing with personal factors for a more comprehensive assessment of active travels practice and frequency.

From a research perspective, the results also demonstrate the advantage of supplementing existing knowledge from the literature and experts opinions, with direct recourse to the population in order to identify new factors. If the approach remains qualitative, text analysis provides methods for identifying the salient aspects of a text corpus. It therefore reveals what is important for individuals. But the wealth of results from these methods can be evaluated only by using a complementary quantitative approach. This allows us to precisely determine the actual roles and weights of these new factors for understanding active mobility behaviours.

This quantitative approach is implemented by the QEVIC questionnaire (S1 Appendix). It was designed for adults on the basis of this study, and it integrates not only the traditional factors of the principal existing questionnaires but also those updated here. It comprises several sections. Following general questions about residence, there are questions about the location, frequentation and travel to activity places for the purpose of accompanying children, leisure, shopping for food and work/studies. These categories of everyday activities, cited in Fig 3, were integrated into the questionnaire to assess the activity space. Work/Studies activity is approached by more thorough questions in connection with transportation infrastructure. The following sections more particularly concern perceptions of the residential neighbourhood and the location where work/studies are carried out, as well as principal transportation modes. Finally, the last two sections relate to transportation modes in the context of their social environment, intentions as regards active travel, criteria for selecting the residential area, and the way in which individuals organize their time in daily life. The organization of time is assessed by taking into account time scheduling between home and other activity places. This directly impacts time spent outside and daily travel time. It is also integrated as the minimization of travel time (S1 Appendix, p. 18).

This questionnaire can be used to simultaneously collect various types of qualitative and quantitative information that can potentially influence active transportation behaviors. Moreover, it is possible to geolocate visited places by using the names of towns and neighborhoods given by the subjects, and thus associate subjective information from questionnaires to objective information on living environments (land use for example). The QEVIC questionnaire is currently used as part of ACTI-Cités research project, which deals with the relationship between environment and the practice of physical activity, which is assessed by another questionnaire [30].

### Current knowledge

Socio-ecological approaches suggest examining behaviour from multiple aspects [31] according to different types of factors (personal, social environment, built environment and institutional). The results of this study support this conceptual approach, but show that it is necessary i) to expand the conception of built environment to the activity space and thus to better describe all transportation systems, and ii) to further integrate, with individual factors, how transportation modes are perceived and the ways in which individuals organize their time. For example, the activity-based approach indicates that some activities are fixed in space and time (work/studies, group activities, etc.), and that these three factors (fixed activities/space/time) moreover form a potential basis to organize the timetable, in particular taking account of the transportation modes available [24]. The results of this study are also supported by other research indicating that i) the living environment cannot be limited to the residential neighborhood and must be extended to the activity space, ii) the organization of time is a fundamental dimension to understanding behaviors [32].

Some questionnaires quoted in this article lead towards expanding the concept of activity space, and take account of how modes of travel are perceived to study active mobility. This is true for the TPAQ [33], which integrates a transportation approach (reason for travel, description of travel and perception of transportation modes), but also for the NN9 [34], which associates journeys with various types of activities to connect them with needs related to walking.

### Strengths and limitations

This study clearly shows that a multi-level approach remains an important and fundamental guide to the comprehension of behaviours. Not only does this study redefine the consideration of certain factors in the built environment (residential neighbourhood versus activity space), but it also shows that within the framework of active mobility, it is essential to supplement individual data by taking account of how time is allocated to activities.

The questionnaires used to study the relationships between the environment (built and social) and active mobility have not changed much over the last 10 years. The NEWS questionnaire, one of the most common and most often used, was discussed in a publication in 2003 [35]. This stability, which is essential for comparing results between studies and over time, should not, however, come at the expense of the evolution of tools required by the dynamic nature of society and of the occupation of urban space—a space which evokes town planning policy and the local context of the real estate market.

This study thus offers a new way to improve our understanding of active mobility behaviours by exploring new factors which are potentially relevant, by means of the QEVIC questionnaire. The QEVIC makes it possible to collect data on living environments (built and social), on travel undertaken by individuals for various reasons as part of daily life (home, work, purchases, leisure and children), on how modes of travel are perceived and on how individuals organize their time for activities.

Within the framework of this study, five aspects must be taken into account in future research on the active mobility of a broad population of adults. First, the typology being based solely on individual statements, these statements could not be compared with the actual practices. Second, this pilot study was conducted to reveal factors that may potentially influence active mobility. But these factors should now be investigated by a complementary quantitative study using the QEVIC in order to determine their influence, as well as their respective weights. Third, one can reasonably assume that a more significant number of interviews would have led to a more marked typology, in which factors with a too low frequency could have constituted a separate class. A greater number of interviews could certainly also reveal more factors, but it is likely that we should significantly increase the sample size to achieve such a result, as stipulated by the Law of large numbers. In our opinion, our typology factors should be generalizable to populations with similar lifestyles, but this does not mean that all people are affected alike by these factors. However, this generalizability should be confirmed in a subsequent study with a larger and more diversified sample. Fourth, the fact that the corpus lacks factors reflecting social relationships, community life and crime in the neighbourhood is due to psychological reasons. It is extremely difficult to spontaneously obtain information about negative facts (such as social) which may question the self-image an interviewee projects. Fifth, the sample only includes residents of a metropolitan area with outlying areas, not residents of remote rural spaces like some hamlets or small villages.

## Conclusions

This study identifies several factors underestimated in previous studies on active mobility that should be integrated simultaneously into new study questionnaires for an adult population. The main factors are: the place of residence within the urban structure, the perception of transportation modes, the whole activity space beyond neighbourhood (including its physical and social environmental aspects), and the organization of time underlying the realization of daily activities, in particular travel. The QEVIC questionnaire, a new tool to study active mobility behaviours, is the result of this research.

## Supporting Information

S1 Appendix QEVIC questionnaire. (PDF)

S2 Appendix Interview guide. (PDF)

S3 Appendix Literature search strategy. (PDF)

S4 Appendix Questionnaires names and reviewed articles. (PDF)

S5 Appendix PRISMA Checklist. (PDF)
