# Supplementary material for: Active Mobility and Environment: A Pilot Qualitative Study for the Design of a New Questionnaire
Source: PLoS One. 2017 Jan 4;12(1):e0168986. doi: 10.1371/journal.pone.0168986 (PMC5215579; doi:10.1371/journal.pone.0168986)
Supplement: S1 Appendix — (PDF) [file pone.0168986.s001.pdf]

QEVIC

Questionnaire on daily life environment

ACTI-Cités Project

This version is provided for information purpose only. Originally in French, the English translation has not been validated. The layout also differs from the web version.

Questionnaire sections :

1. Introduction
2. General issues
3. Activity places
4. Places perception
5. Neighborhoods perception
6. Transportation modes perception
7. Your entourage
8. Opinions about your environment and your organization
9. Comments on the questionnaire

1. Introduction

Research on the links between diet, physical activity and health highlight the importance of the daily frequented places and transportation modes characteristics. This questionnaire has been developed to better understand the characteristics of the places where you live (residence neighborhood, area around the workplace).

This information is very important because it allows researchers to better understand the relationships between physical activity and dietary behaviors, places and health.

Thank you for the time you spend to fill this questionnaire!

2. General issues

Choose the proposal that best fits your main housing

□ In a house
□ In a small building (maximum 3 floors)
□ In a building
□ In a large tower

Choose the proposal that best fits to the location of your home

□ In the center of a village
□ In a subdivision of a village
□ In the center of a small town
□ In a subdivision of a small town
□ In the center of a big city
□ In a neighborhood located between the center of a large city and its suburbs
□ In the suburb of a large city
□ Your home is isolated, or in a hamlet

How much time do you spend on average per week on your place of residence (including nights and weekends), knowing that a week has 168 hours?

☐ hours

Do you own a secondary home?

□ Yes
□ No

On average, how much time do you spend in this home per year?

☐ month

□ Less than a month

3. Activity places

In the everyday life, you move away from home for various reasons. We will ask you to describe your main activity places and transportation modes.

Do you regularly accompany a child or children in places such as the nursery, school or gym?

□ Yes
□ No

→ We would like to have information on the frequency and the transportation modes you use to go to the most usual place to accompany a child.

What is the name of the city where this place is located?

[ ]

What is the name of the neighborhood where this place is located? (if you live in a big city, this will allow us to better locate that place)

[ ]

How many times a week do you go there?

□ Less than once a week
□ 1 or 2 a week
□ 3 to 5 a week
□ More than 5 a week

Which transportation mode(s) do you use ? (multiple choice)

□ Car
□ Motorcycle
□ Public transport
□ Walk
□ Bike
□ Other

If other, please specify: [ ]

What is the main reason why you use this or these modes?

□ The fastest
□ The cheapest
□ No public transport
□ The trip is pleasant
□ I want to be physically active
□ Presence of parking lots
□ Presence of suitable facilities (bike path, footpath)
□ No motorized vehicle or bicycle
□ Other

How much time do you spend on average to go to this place?

[ ] Minutes

Do you have regular leisure activities such as going to the cinema, to the theater, to a concert, in a media library?

□ Yes
□ No

→ We would like to have information on the frequency and the transportation modes you use to go to the most usual place for leisure.

What is the name of the city where this place is located?

[ ]

What is the name of the neighborhood where this place is located? (if you live in a big city, this will allow us to better locate that place)

[ ]

How many times a week do you go there?

□ Less than once a week
□ 1 or 2 a week
□ 3 to 5 a week
□ More than 5 a week

Which transportation mode(s) do you use? (multiple choice)

□ Car
□ Motorcycle
□ Public transport
□ Walk
□ Bike
□ Other

If other, please specify: [ ]

What is the main reason why you use this or these modes?

□ The fastest
□ The cheapest
□ No public transport
□ The trip is pleasant
□ I want to be physically active
□ Presence of parking lots
□ Presence of suitable facilities (bike path, footpath)
□ No motorized vehicle or bicycle
□ Other

How much time do you spend on average to go to this place?

[ ] Minutes

Do you regularly travel (away from home) for your groceries?

- □ Yes
- □ No

→ We would like to have information on the frequency and the transportation modes you use to go to the most usual place for groceries.

What is the name of the city where this place is located?

[ ]

What is the name of the neighborhood where this place is located? (if you live in a big city, this will allow us to better locate that place)

[ ]

How many times a week do you go there?

- □ Less than once a week
- □ 1 or 2 a week
- □ 3 to 5 a week
- □ More than 5 a week

Which transportation mode(s) do you use? (multiple choice)

- □ Car
- □ Motorcycle
- □ Public transport
- □ Walk
- □ Bike
- □ Other

If other, please specify: [ ]

What is the main reason why you use this or these modes?

- □ The fastest
- □ The cheapest
- □ No public transport
- □ The trip is pleasant
- □ I want to be physically active
- □ Presence of parking lots
- □ Presence of suitable facilities (bike path, footpath)
- □ No motorized vehicle or bicycle
- □ Other

How much time do you spend on average to go to this place?

[ ] Minutes

Work/studies

Currently, your workplace or study place is:

□ Fixed (office, factory, school, university...)
□ No fixed workplace (visits…)
□ Work at home
□ Not concerned

What is the name of the city where this place is located?

What is the name of the neighborhood where this place is located? (if you live in a big city, this will allow us to better locate that place)

On average, how much time do you spend per week at your workplace (or study place)?

Hours

How many times a week do you go there?

□ Less than once a week
□ 1 or 2 a week
□ 3 to 5 a week
□ More than 5 a week

Which transportation mode(s) do you use? (multiple choice)

□ Car
□ Motorcycle
□ Public transport
□ Walk
□ Bike
□ Other

If other, please specify:

What is the main reason why you use this or these modes?

□ The fastest
□ The cheapest
□ No public transport
□ The trip is pleasant
□ I want to be physically active
□ Presence of parking lots
□ Presence of suitable facilities (bike path, footpath)
□ No motorized vehicle or bicycle
□ Other

How much time do you spend on average to go to this place?

☐ Minutes

How many vehicles have you got in your household?

Vehicle type	Number of vehicles :				
	0	1	2	3	4 or more
Bike (including those for children)	☐	☐	☐	☐	☐
Motorcycle	☐	☐	☐	☐	☐
Car	☐	☐	☐	☐	☐

Do you have a travelcard for public transport?

☐ Yes
☐ No

Which equipment can you use at your workplace (or study place)? (Multiple choice)

☐ Sports equipment
☐ Changing rooms
☐ Showers
☐ A parking lot for car
☐ A bicycle parking
☐ None of these

Which of the following propositions indicates what is offered by your employer / trainer? (Multiple choice)

☐ Participation for car travel costs
☐ Grants for public transport
☐ Company car
☐ Grants to purchase bicycles
☐ Bike lending
☐ Awareness campaign for the use of other transportation modes than the car
☐ None of the above

4. Places perception

Around your home you have access to: (multiple choice)

□ A small supermarket
□ A grocery store
□ A bank
□ A post office
□ A school
□ A bakery
□ A restaurant
□ A cafe terrace
□ A pharmacy
□ None of the above

Around your workplace you have access to at least: (multiple choice)

□ A small supermarket
□ A grocery store
□ A bank
□ A post office
□ A school
□ A bakery
□ A restaurant
□ A cafe terrace
□ A pharmacy

Around your home, there are:

□ 1 public transport stop
□ 2 public transport stops or more
□ No public transport stop
□ Do not know

If there is at least one transit stop, is the vehicles frequency adapted for your needs?

□ Yes
□ No

Around your workplace, there are:

□ 1 public transport stop
□ 2 public transport stops or more
□ No public transport stop
□ Do not know

If there is at least one transit stop, is the vehicles frequency adapted to your needs?

□ Yes
□ No

Moving around your home

Among the following proposals, choose the one that best describes how you can walk around your home

□ You can walk on the sidewalks without being hindered by other pedestrians, cyclists or parked cars.
□ You can walk on the sidewalks, but you are sometimes hindered by other pedestrians, cyclists or parked cars.
□ On the sidewalks, you are often forced to walk around obstacles or to walk on the road.
□ There are no sidewalks around my home
□ You're not concerned

Among the following proposals, choose the one that best describes how it is possible to move by bike around your home

□ There are bike lanes separated from the road
□ There are bike lanes on roads
□ You must take the road without bike lanes
□ You don't know

Moving around your workplace/study place

Among the following proposals, choose the one that best describes how you can walk around your workplace

□ You can walk on the sidewalks without being hindered by other pedestrians, cyclists or parked cars.
□ You can walk on the sidewalks, but you are sometimes hindered by other pedestrians, cyclists or parked cars.
□ On the sidewalks, you are often forced to walk around obstacles or to walk on the road.
□ There are no sidewalks around my workplace
□ You're not concerned

Among the following proposals, choose the one that best describes how it is possible to move by bike around your workplace

□ There are bike lanes separated from the road
□ There are bike lanes on roads
□ You must take the road without bike lanes
□ You don't know

5. Neighborhoods perception

Is your home neighborhood pleasant?

The neighborhood is polluted

Strongly agree	Agree	Neither agree nor disagree	Disagree	Strongly disagree
☐	☐	☐	☐	☐

The neighborhood is clean and well maintained

Strongly agree	Agree	Neither agree nor disagree	Disagree	Strongly disagree
☐	☐	☐	☐	☐

There are trees along the streets of the neighborhood

Strongly agree	Agree	Neither agree nor disagree	Disagree	Strongly disagree
☐	☐	☐	☐	☐

There is one or more green spaces in the neighborhood

Strongly agree	Agree	Neither agree nor disagree	Disagree	Strongly disagree
☐	☐	☐	☐	☐

There are a lot of poorly maintained, unoccupied or ugly buildings in the neighborhood

Strongly agree	Agree	Neither agree nor disagree	Disagree	Strongly disagree
☐	☐	☐	☐	☐

Do you feel safe in your home neighborhood?

It is not safe to leave a locked bike

Strongly agree	Agree	Neither agree nor disagree	Disagree	Strongly disagree
☐	☐	☐	☐	☐

It is not safe to walk because of road traffic

Strongly agree	Agree	Neither agree nor disagree	Disagree	Strongly disagree
☐	☐	☐	☐	☐

It is not safe to ride a bike because of road traffic

Strongly agree	Agree	Neither agree nor disagree	Disagree	Strongly disagree
☐	☐	☐	☐	☐

During the day, the neighborhood is not safe because of criminality

Strongly agree	Agree	Neither agree nor disagree	Disagree	Strongly disagree
☐	☐	☐	☐	☐

At night, the neighborhood is not safe because of criminality

Strongly agree	Agree	Neither agree nor disagree	Disagree	Strongly disagree
☐	☐	☐	☐	☐

Is your workplace/study place neighborhood pleasant?

The neighborhood is polluted

Strongly agree	Agree	Neither agree nor disagree	Disagree	Strongly disagree
☐	☐	☐	☐	☐

The neighborhood is clean and well maintained

Strongly agree	Agree	Neither agree nor disagree	Disagree	Strongly disagree
☐	☐	☐	☐	☐

There are trees along the streets of the neighborhood

Strongly agree	Agree	Neither agree nor disagree	Disagree	Strongly disagree
☐	☐	☐	☐	☐

There is one or more green spaces in the neighborhood

Strongly agree	Agree	Neither agree nor disagree	Disagree	Strongly disagree
☐	☐	☐	☐	☐

There are a lot of poorly maintained, unoccupied or ugly buildings in the neighborhood

Strongly agree	Agree	Neither agree nor disagree	Disagree	Strongly disagree
☐	☐	☐	☐	☐

Do you feel safe in your workplace/study place neighborhood?

It is not safe to leave a locked bike

Strongly agree	Agree	Neither agree nor disagree	Disagree	Strongly disagree
☐	☐	☐	☐	☐

It is not safe to walk because of road traffic

Strongly agree	Agree	Neither agree nor disagree	Disagree	Strongly disagree
☐	☐	☐	☐	☐

It is not safe to ride a bike because of road traffic

Strongly agree	Agree	Neither agree nor disagree	Disagree	Strongly disagree
☐	☐	☐	☐	☐

During the day, the neighborhood is not safe because of criminality

Strongly agree	Agree	Neither agree nor disagree	Disagree	Strongly disagree
☐	☐	☐	☐	☐

At night, the neighborhood is not safe because of criminality

Strongly agree	Agree	Neither agree nor disagree	Disagree	Strongly disagree
☐	☐	☐	☐	☐

6. Transportation modes perception

Your perception of transportation modes to perform your activities (work, school, shopping, various outings: restaurant, movie theater, visit someone, go to a sports club, etc ...)

For each of the proposed modes, check the box that best fits the way you consider this mode to perform everyday activities (even if you don't use this mode).

Walking is

	Very	Pretty	Not
Fast	☐	☐	☐
Comfortable	☐	☐	☐
Safe, Secure	☐	☐	☐
Convenient	☐	☐	☐
Relaxing	☐	☐	☐

Bike is

	Very	Pretty	Not
Fast	☐	☐	☐
Comfortable	☐	☐	☐
Cheap	☐	☐	☐
Safe, Secure	☐	☐	☐
Convenient	☐	☐	☐
Relaxing	☐	☐	☐
Green	☐	☐	☐

Motorcycle is

	Very	Pretty	Not
Fast	☐	☐	☐
Comfortable	☐	☐	☐
Cheap	☐	☐	☐
Safe, Secure	☐	☐	☐
Convenient	☐	☐	☐
Relaxing	☐	☐	☐
Green	☐	☐	☐

Tram/suburban train/subway is

	Very	Pretty	Not
Fast	☐	☐	☐
Comfortable	☐	☐	☐
Cheap	☐	☐	☐
Safe, Secure	☐	☐	☐
Convenient	☐	☐	☐
Relaxing	☐	☐	☐
Green	☐	☐	☐
Punctual	☐	☐	☐

Bus is

	Very	Pretty	Not
Fast	☐	☐	☐
Comfortable	☐	☐	☐
Cheap	☐	☐	☐
Safe, Secure	☐	☐	☐
Convenient	☐	☐	☐
Relaxing	☐	☐	☐
Green	☐	☐	☐
Punctual	☐	☐	☐

Train is

	Very	Pretty	Not
Fast	☐	☐	☐
Comfortable	☐	☐	☐
Cheap	☐	☐	☐
Safe, Secure	☐	☐	☐
Convenient	☐	☐	☐
Relaxing	☐	☐	☐
Green	☐	☐	☐
Punctual	☐	☐	☐

Car is

	Very	Pretty	Not
Fast	☐	☐	☐
Comfortable	☐	☐	☐
Cheap	☐	☐	☐
Safe, Secure	☐	☐	☐
Convenient	☐	☐	☐
Relaxing	☐	☐	☐
Green	☐	☐	☐

7. Your entourage

The following questions refer to the people around you, the way they move to go to their activity places. We are not interested in the activity itself, but the way to move to the place where the activity is performed.

<u>Your familiy</u>

In your family, most of the adults use bike to reach their activities locations (work, study, shopping, visits, cinema, concert, bar, restaurant, ...)

Strongly agree	Agree	Neither agree nor disagree	Disagree	Strongly disagree
☐	☐	☐	☐	☐

In your family, most of the adults walk to reach their activities locations (work, study, shopping, visits, cinema, concert, bar, restaurant, …)

Strongly agree	Agree	Neither agree nor disagree	Disagree	Strongly disagree
☐	☐	☐	☐	☐

<u>Your friends</u>

Most of your friends use bike to reach their activities locations (work, study, shopping, visits, cinema, concert, bar, restaurant, …)

Strongly agree	Agree	Neither agree nor disagree	Disagree	Strongly disagree
☐	☐	☐	☐	☐

Your friends walk to reach their activities locations (work, study, shopping, visits, cinema, concert, bar, restaurant, …)

Strongly agree	Agree	Neither agree nor disagree	Disagree	Strongly disagree
☐	☐	☐	☐	☐

8. Opinions about your environment and your organization

About your neighborhood, which of the following statements best describes your situation:

□ I want to continue to live in my neighborhood in the future
□ I want to change of neighborhood, and I plan change soon
□ I want to change of neighborhood, but I can't for financial reasons
□ I want to change of neighborhood, but I can't for other reasons (family, work, etc.)
□ No specific wish

What are the main two reasons why you have chosen to live in this neighborhood?

□ Near the workplace or study place
□ For financial reasons
□ The neighborhood is nice
□ Access to public transport is easy
□ Because there are a lot of activities (shops, restaurants)
□ Proximity to green space
□ Because friends and family members living there
□ Other
□ Do not know

If other, please specify :

For the following statements, please specify your level of agreement/disagreement. If you are unable to exercise physical activity, check "not concerned"

On weekdays, I feel out of time to do things.

Strongly agree	Agree	Neither agree nor disagree	Disagree	Strongly disagree
☐	☐	☐	☐	☐

☐ Not concerned

On weekdays, I organize myself to spend as much time as possible at home

Strongly agree	Agree	Neither agree nor disagree	Disagree	Strongly disagree
☐	☐	☐	☐	☐

☐ Not concerned

On weekdays, I organize myself to minimize my travel time.

Strongly agree	Agree	Neither agree nor disagree	Disagree	Strongly disagree
☐	☐	☐	☐	☐

☐ Not concerned

On weekdays, my environment would allow me to walk more often to go to my activities places

Strongly agree	Agree	Neither agree nor disagree	Disagree	Strongly disagree
☐	☐	☐	☐	☐

☐ Not concerned

On weekdays, my environment would allow me to use my bike more often to go to my activities places

Strongly agree	Agree	Neither agree nor disagree	Disagree	Strongly disagree
☐	☐	☐	☐	☐

☐ Not concerned

On weekdays the physical effort required by walking trips is a constraint.

Strongly agree	Agree	Neither agree nor disagree	Disagree	Strongly disagree
☐	☐	☐	☐	☐

☐ Not concerned

On weekdays the physical effort required by cycling trips is a constraint.

Strongly agree	Agree	Neither agree nor disagree	Disagree	Strongly disagree
☐	☐	☐	☐	☐

□ Not concerned

On weekdays, I do not intend to walk more

Strongly agree	Agree	Neither agree nor disagree	Disagree	Strongly disagree
□	□	□	□	□

□ Not concerned

On weekdays, I don't want to use my bike more often

Strongly agree	Agree	Neither agree nor disagree	Disagree	Strongly disagree
□	□	□	□	□

□ Not concerned

9. Comments on the questionnaire

We invite you to give us additional information that will allow us to better treat your answers

____

____

____

____

____

____

____

____
