# Supplementary material for: Active Mobility and Environment: A Pilot Qualitative Study for the Design of a New Questionnaire
Source: PLoS One. 2017 Jan 4;12(1):e0168986. doi: 10.1371/journal.pone.0168986 (PMC5215579; doi:10.1371/journal.pone.0168986)
Supplement: S2 Appendix — (PDF) [file pone.0168986.s002.pdf]

Interview guide

(translated from french for this document)

General instruction of the interview

Thank you for agreeing to participate in this research. I remind you that this interview focuses on the everyday life activities, except those of your intimate life.
I would like you to tell me about the activities and trips that punctuate your days, by describing the places where they take place, the times in the week, the people who are with you, and what motivates or hinders their realization.
For your convenience, I suggest you to talk successively about the activities at home, the activities carried out in your neighbourhood, and then at your city and region scale.

The following guide includes the 5 essential dimensions covered in the interviews. **The questions have not been explicitly asked to individuals during the interview, except as reminders when the point was not naturally addressed by him.**

For each activity mentioned

A) **Places:** Where do you practice this activity?
B) **Time:** When do you practice this activity?
C) **Involved persons**: Are there people with you when you practice this activity?
D) **Motivations and barriers:** What motivates / hinders the practice of this activity?
E) **Trips:** How do you travel to this activity place?
