# Supplementary material for: Active Mobility and Environment: A Pilot Qualitative Study for the Design of a New Questionnaire
Source: PLoS One. 2017 Jan 4;12(1):e0168986. doi: 10.1371/journal.pone.0168986 (PMC5215579; doi:10.1371/journal.pone.0168986)
Supplement: S3 Appendix — (PDF) [file pone.0168986.s003.pdf]

Literature search strategy

Full list of keywords

Thematic keywords list: “physical environment”, “built environment”, “social environment”, “active mobility”, “active transportation”, “walking”, “cycling”, “bicycling”

European Countries keywords list: “Austria”, “Belgium”, “Bulgaria”, “Croatia”, “Cyprus”, “Czech Republic”, “Denmark”, “Estonia”, “Finland”, “France”, “Germany”, “Greece”, “Hungary”, “Ireland”, “Italy”, “Latvia”, “Lithuania”, “Luxembourg”, “Malta”, “Netherlands”, “Poland”, “Portugal”, “Romania”, “Slovakia”, “Slovenia”, “Spain”, “Sweden”, “United Kingdom”.
Also included: “Switzerland”, “Norway”.

Publication date filter: from 2009/01/01 to 2015/05/31

Full requests in databases

PubMed database request:

1) Search in *All Fields*
2) Search ((“physical environment” or “built environment” or “social environment”) AND (“active mobility” or “active transportation” or “walking” or “cycling” or “bicycling”)) AND (“Austria” or “Belgium” or “Bulgaria” or “Croatia” or “Cyprus” or “Czech Republic” or “Denmark” or “Estonia” or “Finland” or “France” or “Germany” or “Greece” or “Hungary” or “Ireland” or “Italy” or “Latvia” or “Lithuania” or “Luxembourg” or “Malta” or “Netherlands” or “Poland” or “Portugal” or “Romania” or “Slovakia” or “Slovenia” or “Spain” or “Sweden” or “United Kingdom” or “Switzerland” or “Norway”) AND ("2009/01/01"[PDat] : "2015/05/31"[PDat])

Web Of Science database request:

1) Search in *TOPIC* field
2) TOPIC: ((“physical environment” or “built environment” or “social environment”) AND (“active mobility” or “active transportation” or “walking” or “cycling” or “bicycling”)) AND (“Austria” or “Belgium” or “Bulgaria” or “Croatia” or “Cyprus” or “Czech Republic” or “Denmark” or “Estonia” or “Finland” or “France” or “Germany” or “Greece” or “Hungary” or “Ireland” or “Italy” or “Latvia” or “Lithuania” or “Luxembourg” or “Malta” or “Netherlands” or “Poland” or “Portugal” or “Romania” or “Slovakia” or “Slovenia” or “Spain” or “Sweden” or “United Kingdom” or “Switzerland” or “Norway”)
   Refined by: PUBLICATION YEARS: (2014 OR 2009 OR 2015 OR 2013 OR 2012 OR 2011 OR 2010)

TRID database request:

1) Search in *Keywords* field
2) ((“physical environment” or “built environment” or “social environment”) AND (“active mobility” or “active transportation” or “walking” or “cycling” or “bicycling”)) AND (“Austria” or “Belgium” or “Bulgaria” or “Croatia” or “Cyprus” or “Czech Republic” or “Denmark” or “Estonia” or “Finland” or “France” or “Germany” or “Greece” or “Hungary” or “Ireland” or “Italy” or “Latvia” or “Lithuania” or “Luxembourg” or “Malta” or “Netherlands” or “Poland” or “Portugal” or “Romania” or “Slovakia” or “Slovenia” or “Spain” or “Sweden” or “United Kingdom” or “Switzerland” or “Norway”)
   Data range: from 200901 to 201505
