# Supplementary material for: Active Mobility and Environment: A Pilot Qualitative Study for the Design of a New Questionnaire
Source: PLoS One. 2017 Jan 4;12(1):e0168986. doi: 10.1371/journal.pone.0168986 (PMC5215579; doi:10.1371/journal.pone.0168986)
Supplement: S4 Appendix — (PDF) [file pone.0168986.s004.pdf]

QUESTIONNAIRES NAMES and REVIEWED ARTICLES (n=54)

**ALPHA (n=3)**<br><br>Link to the questionnaire	1. Spittaels H, Verloigne M, Gidlow C, Gloanec J, Titze S, Foster C, Oppert J-M, Rutter H, Oja P, Sjöström M: **Measuring physical activity-related environmental factors: reliability and predictive validity of the European environmental questionnaire ALPHA**. *International Journal of Behavioral Nutrition and Physical Activity* 2010, **7**:48.<br><br>2. Wallmann B, Spittaels H, De Bourdeaudhuij I, Froboese I: **The perception of the neighborhood environment changes after participation in a pedometer based community intervention**. *Int J Behav Nutr Phys Act* 2012, **9**:33.<br><br>3. Wallmann-Sperlich B, Froboese I, Schantz P: **Physical Activity and the Perceived Neighbourhood Environment—Looking at the Association the Other Way Around**. *International journal of environmental research and public health* 2014, **11**:8093–8111.
**C4T (n=1)**<br><br>Link to the article describing the questionnaire	1. Stronegger WJ, Titze S, Oja P: **Perceived characteristics of the neighborhood and its association with physical activity behavior and self-rated health**. *Health & place* 2010, **16**:736–743.
**IPS/IPAQ-E/PANES (n=6)**<br>Link to the questionnaire	1. Ferrão MM, Gama A, Marques VR, Mendes LL, Mourão I, Nogueira H, Velásquez-Melendez G, Padez C: **Association between parental perceptions of residential neighbourhood environments and childhood obesity in Porto, Portugal**. *European journal of public health* 2013, **23**:1027–1031.<br><br>2. Mota J, Santos R, Pereira M, Teixeira L, Santos MP: **Perceived neighbourhood environmental characteristics and physical activity according to socioeconomic status in adolescent girls**. *Annals of human biology* 2011, **38**:1–6.<br><br>3. Oliveira A, Mota J, Moreira C, Vale S, Abreu S, Moreira P, Santos RM: **Adolescents' perception of environmental features and its association with physical activity: results from de Azorean Physical Activity and Health Study II**. *J Phys Act Health* 2014, **11**:917–921.<br><br>4. Rodríguez-Romo G, Garrido-Muñoz M, Lucía A, Mayorga JI, Ruiz JR: **Association between the characteristics of the neighborhood environment and physical activity**. *Gaceta Sanitaria* 2013, **27**:487–493.<br><br>5. Santos MR, Vale MS, Miranda L, Mota J: **Socio-demographic and perceived environmental correlates of walking in Portuguese adults—A multilevel analysis**. *Health & place* 2009, **15**:1094–1099.<br><br>6. Wallmann B, Bucksch J, Froboese I: **The association between physical activity and perceived environment in German adults**. *The European Journal of Public Health* 2012, **22**:502–508.
**NEWS (n=20)**<br>Link to the questionnaire	1. Arvidsson D, Kawakami N, Ohlsson H, Sundquist K: **Physical activity and concordance between objective and perceived walkability**. *Med Sci Sports Exerc* 2012, **44**:280–7.<br><br>2. Cerin E, Cain KL, Conway TL, Van Dyck D, Hinckson E, Schipperijn J, De Bourdeaudhuij I, Owen N, Davey RC, Hino AAF: **Neighborhood environments and objectively measured physical activity in 11 countries**. *Med Sci Sports Exerc* 2014, **46**:2253–64.<br><br>3. Cerin E, Conway TL, Cain KL, Kerr J, De Bourdeaudhuij I, Owen N, Reis RS, Sarmiento OL, Hinckson EA, Salvo D: **Sharing good NEWS across the world: developing comparable scores across 12 countries for the Neighborhood Environment Walkability Scale (NEWS)**. *BMC Public Health* 2013, **13**:309.<br><br>4. De Bourdeaudhuij I, Van Dyck D, Salvo D, Davey R, Reis RS, Schofield G, Sarmiento OL, Mitas J, Christiansen LB, MacFarlane D: **International study of perceived neighbourhood environmental attributes and Body Mass Index: IPEN Adult study in 12 countries**. *International Journal of Behavioral Nutrition and Physical Activity* 2015, **12**:62.<br><br>5. Deforche B, Van Dyck D, Verloigne M, De Bourdeaudhuij I: **Perceived social and physical environmental correlates of physical activity in older adolescents and the moderating effect of self-efficacy**. *Preventive medicine* 2010, **50**:S24–S29.

	6. Dewulf B, Neutens T, Van Dyck D, De Bourdeaudhuij I, Van de Weghe N: **Correspondence between objective and perceived walking times to urban destinations: Influence of physical activity, neighbourhood walkability, and socio-demographics**. *Int J Health Geogr* 2012, **11**:43.<br><br>7. Kaczynski AT, Glover TD: **Talking the talk, walking the walk: examining the effect of neighbourhood walkability and social connectedness on physical activity**. *Journal of public health* 2012:fds011.<br><br>8. Mäki-Opas TE, de Munter J, Maas J, Hertog F den, Kunst AE: **The association between physical environment and cycling to school among Turkish and Moroccan adolescents in Amsterdam**. *International journal of public health* 2014, **59**:629–636.<br><br>9. Nelson NM, Woods CB: **Neighborhood perceptions and active commuting to school among adolescent boys and girls**. *Journal of physical activity & health* 2010, **7**:257.<br><br>10. Owen N, De De Bourdeaudhuij I, Sugiyama T, Leslie E, Cerin E, Van Van Dyck D, Bauman A: **Bicycle use for transport in an Australian and a Belgian city: associations with built-environment attributes**. *Journal of urban health* 2010, **87**:189–198.<br><br>11. Rottmann M, Mielck A: **['Walkability'and physical activity-results of empirical studies based on the'Neighbourhood Environment Walkability Scale (NEWS)'].** *Gesundheitswesen (Bundesverband der Arzte des Offentlichen Gesundheitsdienstes (Germany))* 2014, **76**:108–115.<br><br>12. Sugiyama T, Cerin E, Owen N, Oyeyemi AL, Conway TL, Van Dyck D, Schipperijn J, Macfarlane DJ, Salvo D, Reis RS: **Perceived neighbourhood environmental attributes associated with adults' recreational walking: IPEN Adult study in 12 countries**. *Health & place* 2014, **28**:22–30.<br><br>13. Van Cauwenberg J, De Bourdeaudhuij I, De Meester F, Van Dyck D, Salmon J, Clarys P, Deforche B: **Relationship between the physical environment and physical activity in older adults: a systematic review**. *Health & place* 2011, **17**:458–469.<br><br>14. Van Dyck D, Cardon G, Deforche B, Giles-Corti B, Sallis JF, Owen N, De Bourdeaudhuij I: **Environmental and psychosocial correlates of accelerometer-assessed and self-reported physical activity in Belgian adults**. *International journal of behavioral medicine* 2011, **18**:235–245.<br><br>15. Van Dyck D, Cerin E, Cardon G, Deforche B, Sallis JF, Owen N, De Bourdeaudhuij I: **Physical activity as a mediator of the associations between neighborhood walkability and adiposity in Belgian adults**. *Health & place* 2010, **16**:952–960.<br><br>16. Van Dyck D, Cerin E, Conway TL, De Bourdeaudhuij I, Owen N, Kerr J, Cardon G, Frank LD, Saelens BE, Sallis JF: **Associations between perceived neighborhood environmental attributes and adults' sedentary behavior: findings from the USA, Australia and Belgium**. *Social science & medicine* 2012, **74**:1375–1384.<br><br>17. Van Dyck D, Cerin E, Conway TL, De Bourdeaudhuij I, Owen N, Kerr J, Cardon G, Frank LD, Saelens BE, Sallis JF: **Perceived neighborhood environmental attributes associated with adults' transport-related walking and cycling: Findings from the USA, Australia and Belgium**. *Int J Behav Nutr Phys Act* 2012, **9**:1–14.<br><br>18. Van Dyck D, Cerin E, Conway TL, De Bourdeaudhuij I, Owen N, Kerr J, Cardon G, Frank LD, Saelens BE, Sallis JF: **Perceived neighborhood environmental attributes associated with adults' leisure-time physical activity: findings from Belgium, Australia and the USA**. *Health & place* 2013, **19**:59–68.<br><br>19. Van Dyck D, De Meester F, Cardon G, Deforche B, De Bourdeaudhuij I: **Physical environmental attributes and active transportation in Belgium: what about adults and adolescents living in the same neighborhoods?** *Health Promotion* 2013, **27**:330–338.<br><br>20. Van Kann DHH, Kremers SPJ, Gubbels JS, Bartelink NHM, De Vries SI, de Vries NK, Jansen MWJ: **The association between the physical environment of primary schools and active school transport**. *Environment and Behavior* 2014:0013916513519644.
**NPAQ (n=1)**<br>Link to the questionnaire	1. Van Dyck D, Cardon G, Deforche B, De Bourdeaudhuij I: **Urban–rural differences in physical activity in Belgian adults and the importance of psychosocial factors**. *Journal of Urban Health* 2011, **88**:154–167.
**PENS (n=1)**<br>Link to the questionnaire	1. Adams EJ, Goodman A, Sahlqvist S, Bull FC, Ogilvie D: **Correlates of walking and cycling for transport and recreation: factor structure, reliability and behavioural associations of the**

	**perceptions of the environment in the neighbourhood scale (PENS)**. *Int J Behav Nutr Phys Act* 2013, **10**.
**TPAQ (n=1)** Link to the questionnaire	1. Adams EJ, Goad M, Sahlqvist S, Bull FC, Cooper AR, Ogilvie D, Consortium iConnect: **Reliability and Validity of the Transport and Physical Activity Questionnaire (TPAQ) for Assessing Physical Activity Behaviour**. 2014.
**NN1 (n=1)** Link to the article	1. Van Dyck D, Cardon G, Deforche B, Owen N, De Bourdeaudhuij I: **Relationships between neighborhood walkability and adults' physical activity: How important is residential self-selection?** *Health & place* 2011, **17**:1011–1014.
**NN2 (n=1)** Link to the items	1. Van Dyck D, Veitch J, De Bourdeaudhuij I, Thornton L, Ball K: **Environmental perceptions as mediators of the relationship between the objective built environment and walking among socio-economically disadvantaged women**. *Int J Behav Nutr Phys Act* 2013, **10**:10.1186.
**NN3 (n=1)** Link to the article	1. Van Cauwenberg J, De Donder L, Clarys P, De Bourdeaudhuij I, Buffel T, De Witte N, Dury S, Verté D, Deforche B: **Relationships between the perceived neighborhood social environment and walking for transportation among older adults**. *Social Science & Medicine* 2014, **104**:23–30.
**NN4 (n=1)** Link to the article	1. Van Cauwenberg J, Clarys P, De Bourdeaudhuij I, Van Holle V, Verté D, De Witte N, De Donder L, Buffel T, Dury S, Deforche B: **Physical environmental factors related to walking and cycling in older adults: the Belgian aging studies**. *BMC public health* 2012, **12**:142.
**NN5 (n=1)** Link to the article	1. Van Cauwenberg J, Clarys P, De Bourdeaudhuij I, Van Holle V, Verté D, De Witte N, De Donder L, Buffel T, Dury S, Deforche B: **Older adults' transportation walking: a cross-sectional study on the cumulative influence of physical environmental factors**. *International journal of health geographics* 2013, **12**:37–37.
**NN6 (n=1)** Link to the article	1. Toftager M, Ekholm O, Schipperijn J, Stigsdotter U, Bentsen P, Gronbaek M, Randrup TB, Kamper-Jorgensen F: **Distance to green space and physical activity: a Danish national representative survey**. *J Phys Act Health* 2011, **8**:741–749.
**NN7 (n=1)** Link to the items	1. Panter J, Corder K, Griffin SJ, Jones AP, van Sluijs EM: **Individual, socio-cultural and environmental predictors of uptake and maintenance of active commuting in children: longitudinal results from the SPEEDY study**. *Int J Behav Nutr Phys Act* 2013, **10**:83.
**NN8 (n=1)** Link to the article	1. Nevelsteen K, Steenberghen T, Van Rompaey A, Uyttersprot L: **Controlling factors of the parental safety perception on children's travel mode choice**. *Accident Analysis & Prevention* 2012, **45**:39–49.
**NN9 (n=1)** Link to the article	1. Lindelöw D, Svensson Å, Sternudd C, Johansson M: **What limits the pedestrian? Exploring perceptions of walking in the built environment and in the context of every-day life**. *Journal of Transport & Health* 2014, **1**:223–231.
**NN10 (n=1)** Link to the article	1. Kamphuis CB, Mackenbach JP, Giskes K, Huisman M, Brug J, Van Lenthe FJ: **Why do poor people perceive poor neighbourhoods? The role of objective neighbourhood features and psychosocial factors**. *Health & place* 2010, **16**:744–754.
**NN11 (n=1)** Link to the article	1. Haybatollahi M, Czepkiewicz M, Laatikainen T, Kyttä M: **Neighbourhood preferences, active travel behaviour, and built environment: an exploratory study**. *Transportation research part F: traffic psychology and behaviour* 2015, **29**:57–69.
**NN12 (n=1)** Link to the article	1. Gidlow C, Cochrane T, Davey RC, Smith G, Fairburn J: **Relative importance of physical and social aspects of perceived neighbourhood environment for self-reported health**. *Preventive medicine* 2010, **51**:157–163.
**NN13 (n=1)** Link to the article	1. Engbers LH, Hendriksen IJ: **Characteristics of a population of commuter cyclists in the Netherlands: perceived barriers and facilitators in the personal, social and physical environment**. *Int J Behav Nutr Phys Act* 2010, **7**:89–93.
**NN14 (n=1)** Link to the article	1. Aarts M-J, Mathijssen JJ, van Oers JA, Schuit AJ: **Associations between environmental characteristics and active commuting to school among children: a cross-sectional study**. *International journal of behavioral medicine* 2013, **20**:538–555.
**NN15 (n=2)** Link to the article	1. Ducheyne F, De Bourdeaudhuij I, Lenoir M, Cardon G: **Test-retest reliability and validity of a child and parental questionnaire on specific determinants of cycling to school**. *Pediatric exercise science* 2012, **24**:289.<br>2. Ducheyne F, De Bourdeaudhuij I, Spittaels H, Cardon G: **Individual, social and physical environmental correlates of "never"and "always" cycling to school among 10 to 12 year old**

	**children living within a 3.0 km distance from school**. *International journal of behavioral nutrition and physical activity* 2012, **9**:142.
**NN16 (n=1)** Link to the article	1. Aditjandra P, Mulley C, Nelson J: **Extent to Which Sustainable Travel to Work Can Be Explained by Neighborhood Design Characteristics**. *Transportation Research Record: Journal of the Transportation Research Board* 2009:114–122.
**NN17 (n=1)** Link to the article	1. Carver A, Panter JR, Jones AP, van Sluijs EM: **Independent mobility on the journey to school: A joint cross-sectional and prospective exploration of social and physical environmental influences**. *Journal of transport & health* 2014, **1**:25–32.
**NN18 (n=1)** Link to the article	1. Christiansen LB, Toftager M, Schipperijn J, Ersbøll AK, Giles-Corti B, Troelsen J: **School site walkability and active school transport–association, mediation and moderation**. *Journal of transport geography* 2014, **34**:7–15.
**NN19 (n=1)** Link to the article	1. Van Acker V, Derudder B, Witlox F: **Why people use their cars while the built environment imposes cycling**. *Journal of Transport and Land Use* 2013, **6**:53–62.
**NN20 (n=1)** Link to the article	1. Molina-García J, Castillo I, Sallis JF: **Psychosocial and environmental correlates of active commuting for university students**. *Preventive medicine* 2010, **51**:136–138.
